# Molecular Epidemiology of Hepatitis E Virus in Hungary (2018–2025): Emergence of Rare Subtypes and First Detection of HEV-4 in Central Europe

**DOI:** 10.3390/v17101389

**Published:** 2025-10-18

**Authors:** Ágnes Dencs, Andrea Hettmann, Levente Zsichla, Viktor Müller, Anett Dömötör, Ágnes Barna-Lázár, Erzsébet Barcsay, Mária Takács

**Affiliations:** 1 National Reference Laboratory for Hepatitis Viruses, National Center for Public Health and Pharmacy, 1437 Budapest, Hungary; dencs.agnes@nngyk.gov.hu (Á.D.); hettmann.andrea@nngyk.gov.hu (A.H.); domotor.anett@nngyk.gov.hu (A.D.); barnalazar.agnes@nngyk.gov.hu (Á.B.-L.); barcsay.erzsebet@nngyk.gov.hu (E.B.); 2 Institute of Biology, ELTE Eötvös Loránd University, 1117 Budapest, Hungary; zsichla.levente@ttk.elte.hu (L.Z.); mueller.viktor@ttk.elte.hu (V.M.); 3 National Laboratory for Health Security, ELTE Eötvös Loránd University, 1117 Budapest, Hungary; 4 Institute of Medical Microbiology, Semmelweis University, 1089 Budapest, Hungary

**Keywords:** hepatitis E virus, molecular epidemiology, molecular surveillance, genotype, subtype, sequencing, phylogenetic analysis, virus strain

## Abstract

Hepatitis E virus (HEV) is an emerging cause of viral hepatitis in Europe, with increasing recognition in immunocompromised patients. While genotype 3 (HEV-3) is most prevalent in the region, molecular epidemiology data from Hungary have been limited. HEV strains from 118 RNA-positive patients diagnosed between 2018 and 2025 were genotyped. Next-generation sequencing yielded near-complete HEV genomes for 76 samples. HEV-3 was dominant (98.3%). Subtype 3a was the most common (34.7%), followed by 3c, 3f, and 3e. Rare subtypes (3g, 3h, 3i, 3m, 3ra) and HEV-4b were detected for the first time in Hungary. Among immunocompromised patients, 41.6% developed chronic infection. Ribavirin resistance-associated mutations G1634R and V1479I were frequently detected. In silico analysis of potential multiple infections indicated the presence of at least two HEV strains of distinct origin in six patients. Our surveillance revealed extensive genetic diversity of HEV in Hungary. The detection of rare HEV-3 subtypes and the first documented occurrence of HEV-4b in the country highlight likely viral introductions linked to the increasing international trade. Ongoing surveillance is essential in protecting high-risk groups and limiting HEV transmission in a globalized food system.

## 1. Introduction

*Paslahepevirus balayani*, also known as hepatitis E virus (HEV), is classified in the *Hepeviridae* family and is a major cause of acute viral hepatitis worldwide. The World Health Organization (WHO) estimates that the virus caused approximately 19.47 million cases of acute hepatitis in 2021 and was responsible for 5.4% of global disability-adjusted life years related to acute hepatitis [1].

While most HEV infections are asymptomatic or mild, the clinical course can be severe, particularly in elderly patients, immunocompromised individuals, and those with underlying chronic liver disease [2]. There are also reports of extrahepatic manifestations involving both the peripheral and central nervous systems [3].

Eight genotypes of HEV have been described. Genotypes 1 and 2 infect only humans and are transmitted via the fecal-oral route. They primarily cause waterborne outbreaks in low- and middle-income countries in Asia and Africa and are responsible for the majority of overt cases of hepatitis E. Infection with genotype 1 HEV in pregnant women may lead to acute liver failure with a high case fatality rate [4]. Genotype 3 (HEV-3) is found worldwide and is the dominant type in Europe, North and South America, and Australia. In addition to humans, genotype 3 also infects pigs, wild boar, deer, rabbits, goats, and sheep [5]. In humans, it typically causes sporadic zoonotic infections through the consumption of undercooked meat and meat products. Genotype 4 is also zoonotic, with pigs as the primary reservoir. It originates from Asia and is only occasionally detected in developed countries [6,7,8,9]. Genotypes 3 and 4 generally cause milder disease, but while most infections are self-limiting, they can cause chronic infection in immunocompromised patients, particularly in solid organ transplant (SOT) recipients, in those with hematological diseases, and in patients living with human immunodeficiency virus infection. HEV can lead to severe liver damage in chronically infected patients [10]. Reduction in immunosuppression results in viral clearance in one-third of patients chronically infected with HEV [11]. Ribavirin monotherapy is widely used to treat chronic HEV infection, and around 80% of patients achieve sustained viral response (SVR) after three months of therapy. SVR rate may be increased up to 90% using a prolonged treatment course [12].

Genotypes 5 and 6 have been detected in wild boars in Japan, genotype 7 was found in dromedary camels, and genotype 8 in Bactrian camels [13,14,15,16]. Genotype 7 has been found to cause chronic human infection in a liver transplant patient [15]. The genus *Rocahepevirus* (formerly *Orthohepevirus C*) includes *Rocahepevirus ratti*, a rat-derived virus with zoonotic potential that can cause both acute and chronic infections in humans [17,18].

HEV variants can be classified into subtypes within genotypes. A total of 36 subtypes has been proposed, but several genomes remain unassigned until more sequence data become available. HEV-3 subtypes are designated 3a through 3m and are grouped into two major clades: HEV-3efg (Clade 1) and HEV-3abjkchilm (Clade 2). Although no consistent clade-specific differences in pathogenicity or treatment response have been demonstrated, recognizing the two clades provides a useful **basis** for monitoring shifts in the geographical distribution of HEV subtypes. Rabbit-derived HEV strains (3ra) form a distinct group within genotype 3. Genetic distances observed between 3ra genomes will likely warrant their division into several different subtypes [19,20].

Similarly to other viral infections, multiple HEV infections involving different subtypes or strains have occasionally been documented in both immunocompetent and immunocompromised individuals [21,22]. The potential epidemiological and clinical significance of such cases, as well as their frequency, remain largely unassessed; however, multiple infections may provide an opportunity for recombination between HEV strains. Although both laboratory and bioinformatic approaches exist to detect multiple viral infections within hosts, systematic evaluations of this kind have so far been rarely conducted [23,24].

HEV has a single-stranded, positive-sense RNA genome of about 7.2 kb. The largest of its open reading frames, ORF1, comprises several domains, including a polyproline or hypervariable region (PPR or HVR). The length of the PPR depends on viral genotype and subtype, and sequence diversity is higher within zoonotic genotypes, which are adapted to many different hosts [25]. In chronically infected patients, the length of the PPR may also be modified by deletions and insertions. Large deletions in the PPR cause attenuation, while insertions result in improved replication in vitro [26,27]. Insertions originate either from the viral genome, such as duplications of the PPR itself and of the RNA-dependent RNA polymerase (RdRp), or from the host genome. The so far observed human genome insertions into the HEV PPR include sequences encoding human ribosomal proteins S19 and S17, human tyrosine aminotransferase (TAT), human inter-α-trypsin inhibitor (ITI), eukaryotic translation elongation factor (EEF1A1P13), the 18S ribosomal pseudogene (RNA 18SP5), kinesin-like protein KIF1B, and zinc finger protein 787 [28]. The RNA-dependent RNA polymerase is also encoded by ORF1. Current evidence suggests that these events may arise from template switching during replication, which can lead to the incorporation of host or viral sequences. Although some insertions may be random, several studies indicate that they can confer a replication advantage, particularly in chronic infections in immunocompromised patients. Several mutations of the RdRp have been detected in patient-derived HEV isolates and are associated with antiviral therapy failure [29,30]. Some of these mutations, caused by the mutagenic effect of ribavirin, have been shown to increase the replicative capacity of the virus [31].

The distribution of HEV-3 subtypes varies across European countries. Subtype distribution data in several studies show a wide range of circulating variants, but a predominance of subtype 3f from Clade 1 and subtypes 3a and 3c from Clade 2 [32,33,34,35,36,37]. In countries where surveillance has been in place for longer, some information on the dynamics is also available [38,39]. As HEV-3 causes almost exclusively zoonotic infections, the detected subtypes correlate with factors such as food consumption habits and traditions, as well as the trade of meat, meat products, and live animals. In some cases, these result in country-specific monophyletic groups of sequences [40,41]. Imported meat mainly causes sporadic infections; the importation of live pigs poses a greater risk by potentially establishing the sustained circulation of new subtypes.

Given Hungary’s central role in European livestock farming and food trade, characterizing its HEV subtype landscape is particularly important for understanding both national risks and cross-border transmission dynamics. Although hepatitis E virus is endemic in Hungary, information on the circulating subtypes in humans and animals has been limited [42,43,44]. Here we report the results of our country-wide molecular surveillance from 2018 to 2025. The aim of this study was to comprehensively characterize the HEV subtypes circulating in Hungary. Viruses detected from serum samples of patients with acute viral hepatitis were genotyped, and near-complete genomes were sequenced using next-generation sequencing. The surveillance identified a broad spectrum of subtypes, including rare variants and the first documented occurrence of HEV-4 in Central Europe.

## 2. Materials and Methods

### 2.1. Samples

**Figure 1:**
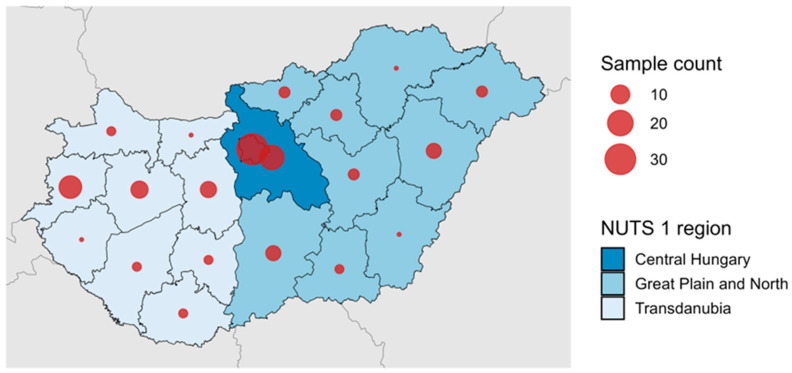
Distribution of genotyped hepatitis E viruses (N = 118). Circle sizes are proportional to the number of samples from each NUTS 3 region. NUTS 1 regions (Transdanubia, Great Plain and North, Central Hungary) are shown in different colors. Place of residence was unknown for 2 patients.

Out of 2013 cases of HEV infections reported across Hungary between 2018 and 2025, 118 RNA-positive samples were available in the reference laboratory for inclusion in the molecular surveillance. The serum samples were received by the reference laboratory for hepatitis E virus testing between April 2018 and May 2025. Samples from all 20 NUTS (Nomenclature of territorial units for statistics) 3 regions of Hungary were included in the surveillance, though Budapest and Pest county were overrepresented, because the laboratory mainly serves the central part of the country for diagnostic purposes (Figure 1). The patients showed symptoms of acute viral hepatitis or had elevated liver enzyme levels discovered as part of routine screening performed for solid organ transplant patients, or before chemotherapy or biological therapy. The median age of the patients was 62.7 (7.0–93.7). Most patients were male (61.9%), and 64 (56.8%) of them were hospitalized. Forty-eight patients were immunocompromised: 35 were SOT patients, 9 had been diagnosed with malignancies, one received methotrexate for rheumatoid arthritis, one had thrombotic thrombocytopenic purpura, and one was a patient with liver cirrhosis, who received biological therapy. Clinical data regarding the indication for biological therapy were not available for one patient. Twenty patients progressed to chronic infection (RNA persistence > 3 months) and received ribavirin therapy. Possible sources of exposure were rarely reported by clinicians, but included the consumption of game meat and pork brawn. Samples were stored at −20 ℃, and isolated nucleic acids were kept at −80 °C until analysis.

### 2.2. Detection and Genotyping of Hepatitis E Virus RNA Based on ORF2

Nucleic acids were isolated from 300 µL sample using Perkin Elmer Chemagic 360 with the chemagic Viral DNA/RNA 300 Kit H96 kit (Perkin Elmer, Product no.: CMG-1033-S, Waltham, MA, USA). Viral RNA was detected and quantified using Altona RealStar^®^ HEV RT-PCR Kit 2.0 (altona Diagnostics GmbH, order no.: 272013, Hamburg, Germany) according to the manufacturer’s instructions. The detected viruses were genotyped based on a 348 bp long segment from ORF2 amplified using a previously published method [45]. Products were visualized on a 2% agarose gel.

PCR products were purified using 0.5 μL Exonuclease I and 1 μL Shrimp Alkaline Phosphatase (Thermo Fisher Scientific, Carlsbad, CA, USA). Sequencing reaction was performed using the BigDye Terminator v3.1 Cycle Sequencing Kit (Thermo Fisher Scientific, Carlsbad, CA, USA, cat. no.: 4337456), purified using the BigDye XTerminator^TM^ Purification Kit and analyzed on an ABI 3500 Series Genetic Analyzer (Thermo Fisher Scientific, Carlsbad, CA, USA). Viruses were genotyped by the RIVM HEVNET genotyping tool (https://www.rivm.nl/mpf/typingtool/hev/, accessed on 27 February 2025) [46].

### 2.3. Illumina Whole-Genome Sequencing

Near-complete genomes were obtained for 76 patients by next-generation sequencing. Samples with viral loads exceeding 30,000 IU/mL were selected, as lower-titer samples did not consistently amplify across all genomic regions. Four overlapping amplicons were generated spanning positions 13 to 7145 on the reference genome prototype 3c strain (FJ705359). Previously published primers were modified based on more recent sequence data available in GenBank and the dominant subtypes determined by the genotyping of ORF2 [47]. One additional primer was designed (2190r). The sequences and positions of the primers used are shown in Table 1.

Reactions were set up using the SuperScript™ III One-Step RT-PCR System with Platinum™ Taq High Fidelity DNA Polymerase (Thermo Fisher Scientific, Carlsbad, CA, USA, cat. no.: 12574035) with 5 µL of isolated RNA and 20 pmol of each primer in 25 µL final volume. The reaction was carried out as follows: 30 min at 53 °C for the RT reaction, 2 min denaturation at 94 °C and 40 cycles of 15 s at 94 °C, 30 s at 60 °C and 4 min at 68 °C. Preparation of amplicon libraries was carried out as described previously [48]. Pooled libraries were sequenced on an Illumina MiSeq sequencer using MiSeq Reagent Micro Kit v2 with 300 cycles (Illumina, San Diego, CA, USA, cat. no.: MS-102-2002). All PPR sequences were confirmed by Sanger sequencing as described above, using primers specific for that region (Table 1).

### 2.4. Mutational and Phylogenetic Analyses

Sequencing reads were trimmed for low-quality bases, adapter and primer sequences, filtered for contaminant reads and assembled using the dshiver genome assembly pipeline (v1.7.3_1.0) with default parameters [49]. Single-nucleotide polymorphisms (SNPs) were identified from the consensus-mapped, processed reads using LoFreq (v2.1.5), and variants with >1% prevalence were retained for further analysis [50]. In addition to the contigs generated by IVA (v1.0.11) within dshiver, we also generated de novo contigs using rnaviralSPAdes, part of the SPAdes package within the Galaxy platform (Galaxy Version 4.2.0+galaxy0) [51,52,53]. The presence or absence of ribavirin (RBV) resistance mutations was detected in both the consensus genomes and the SNP data using custom scripts in R (v4.3.1).

We performed phylogenetic analyses for the 304 bp partial HEV sequences from ORF2 (after removal of primer sequences from the 348 bp product) and near-complete genome sequences (>80% coverage) separately. In addition to the samples generated in this study, representative sequences for genotypes 1–8 were included as suggested by Smith et al. [20]. For each Hungarian sample, the five most similar sequences were also retrieved from GenBank using BLASTn (version 2.12.0) searches against all HEV sequences (Taxonomy ID: 291484). The resulting sequence sets were deduplicated before analysis.

Multiple sequence alignments were performed for the partial and near-complete HEV genome datasets using MAFFT v7 [54]. Maximum likelihood phylogenetic trees were constructed using RAxML-NG v1.2.2 under the GTR+G model [55]. Branch support values were estimated using the automated bootstrap method in RAxML-NG, with a maximum of 500 replicates. The tree based on near-complete genomes was rooted using the subtype 1 sequence M73218.1 as an outgroup. In contrast, the tree constructed from partial genomes was midpoint-rooted, as the phylogenetic placement of the outgroup sequence (Paslahepevirus alci KF951328) was not sufficiently reliable to support confident rooting.

Alignments of PPR amino acid sequences were visualized using BioEdit version 7.7.1 [56].

### 2.5. Detection of Multiple Infections Using Phyloscanner

All 76 deep-sequencing samples were analyzed using phyloscanner (v1.9.3) to infer phylogenetic relationships among all pairs of individuals [23]. We modified the default command-line specification for the first analysis stage to generate genomic windows spanning positions 100 to 7099, with a window length of 140 bp and a step size of 100 bp. We also aligned manually curated reference sequences to the samples using the alignment-of-other-refs flag and specified the GTR+G model for phylogenetic reconstruction via RAxML-NG. In the second analysis stage, we adjusted the default parameters to root the phylogenetic trees using the FJ906895.1 sequence as an outgroup. We set a multifurcation threshold to round any branch corresponding to less than a quarter of a single base change to zero length. Additionally, we allowed for multiple introductions by incorporating a diversity penalty into the parsimony-based state reconstruction, controlled by the parameter k. We explored values of 1, 5, 10, and 12.5, and selected k = 10 as the default. For each sample, a genomic window was considered to have sufficient quality for further analysis if it was covered by at least 100 reads and included at least 10 tips in the corresponding phyloscanner tree.

Based on the results of de novo assembly and phyloscanner summary statistics, we developed three criteria to identify potential multiple infections and distinguish them from sequencing artifacts:

1. De novo assembly criterion: More than one overlapping but genetically distinct contig was assembled from the reads by either IVA or rnaviralSPAdes.
2. Phyloscanner multiple subgraph criterion: In at least 40% of analyzed windows, multiple subgraphs with a prevalence of at least 5% were identified.
3. Phyloscanner diversity criterion: The sample falls within the top two deciles among all samples in both of the following indicators:
  a. Root-to-tip distance indicator: The mean (across all windows) of the difference between (i) the mean patristic distance from all tips from this host to their most recent common ancestor (MRCA), and (ii) the mean patristic distance from the tips within the largest subgraph from the host to the MRCA of that subgraph.
  b. Read patristic distance indicator: The mean (across all windows) of the difference between (i) the mean pairwise patristic distance among all tips from the host, and (ii) the mean pairwise patristic distance among tips within the largest subgraph.

To ensure robustness, samples meeting at least two of the three criteria were considered multiply infected in this study.

### 2.6. Statistical Analysis

Chi-square tests and logistic regression were performed using GraphPad Prism version 10.2.0 for Windows (GraphPad Software, Boston, MA, USA, www.graphpad.com).

### 2.7. Accession Numbers

Near-complete genome sequences and additional ORF2 and partial genome sequences were deposited in GenBank under accession numbers: PV877305-PV877343, PX108670-PX108747 and PX226753-PX226754.

## 3. Results

### 3.1. Detected Genotypes

**Figure 2:**
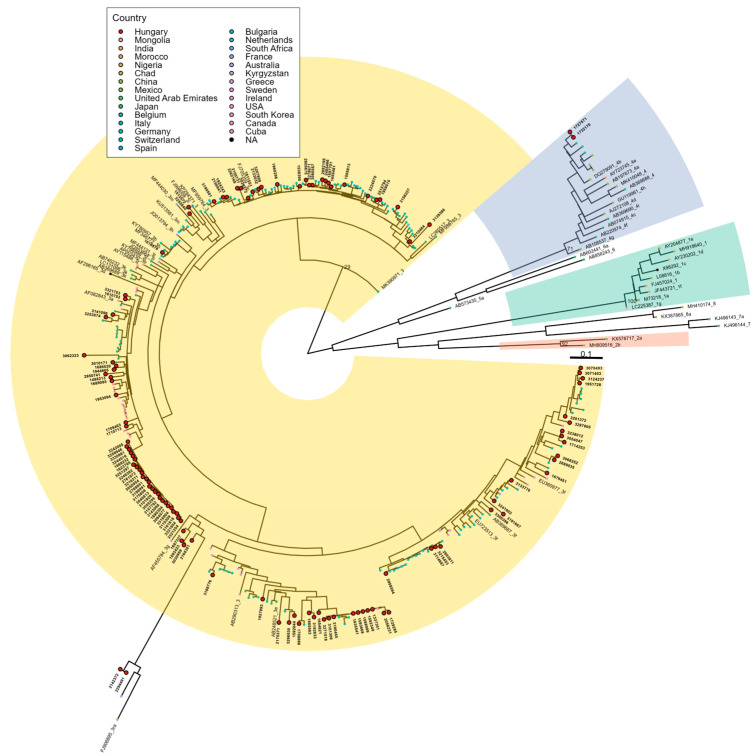
Maximum-likelihood phylogenetic tree of 117 partial capsid protein gene sequences (304 bp) obtained from HEV-positive individuals in Hungary. One partial sequence (PX226754) was not included because it was from a different segment of ORF2. Additionally, the tree includes both a reference dataset [20] and the most similar sequences retrieved from NCBI GenBank. Each tip represents an individual sample, colored according to its origin and samples sequenced in this study are highlighted with enlarged red circles outlined in black. The four major human-infecting HEV clades–genotypes 1, 2, 3, and 4—are highlighted in green, red, yellow, and blue, respectively, with clade branch support values shown. Tree was made using RAxML-NG, rooted at its midpoint and visualized in R version 4.3.1.

HEVNET classified all 118 samples into one of the four main genotypes with high phylogenetic support (≥80%), and subtype classification was strongly supported (>70%) for 67 of 118 partial ORF2 sequences. Of the remaining 51 samples, HEVNET provided subtype assignments for 35 using near-complete genomes, resulting in a total of 102 of 118 samples with highly supported subtyping. Independent phylogenetic analysis using all available partial capsid gene segments, reference genomes and most similar sequences from GenBank confirmed all high-confidence subtype assignments by HEVNET and could also be used to assign subtypes to sequences not subtyped by HEVNET (Figure 2). Among the 16 sequences without assigned subtypes, 15 showed close genetic relationships (genetic distance ≤ 0.12) with previously subtyped sequences and were therefore considered eligible for subtyping [20]. The remaining sequence (sample no.: 3062323) is reported as genotype 3 in this study.

Based on the above analyses, HEV-3 was found to be dominant (116/118, 98.3%) in Hungary. In two patients subtype 4b was found. Out of 116 HEV-3 viruses, 115 were subtyped, and the identified subtypes showed great diversity. The most common subtypes were 3a (41/118, 34.7%), 3c (25/118, 21.2%), 3f (20/118, 16.9%) and 3e (20/118, 16.9%). Together, subtypes 3a, 3c, 3f and 3e accounted for 90% of the cases. A wide range of rarer subtypes were also found: 3g (*n* = 4), 3h (*n* = 1), 3i (*n* = 1), 3m (*n* = 1) and 3ra (*n* = 2). Genotype 1 was not detected in any of the patients.

**Figure 3:**
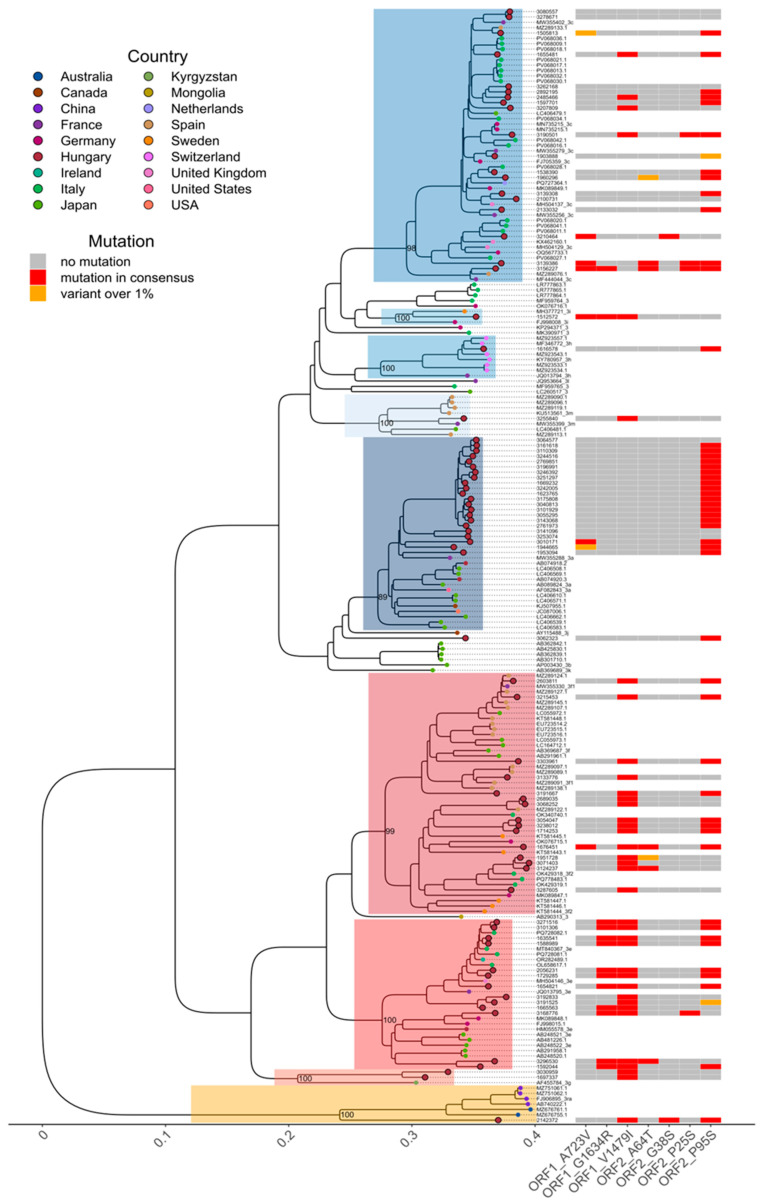
Maximum-likelihood phylogenetic tree and RBV resistance analysis of near-complete HEV genomes from HEV-positive individuals in Hungary. The tree includes both a reference dataset representing major HEV genotype 3 (HEV-3) subtypes as suggested by Smith et al. [20] and most similar sequences retrieved from NCBI GenBank. Each tip represents an individual sample, colored according to its origin and samples sequenced in this study are highlighted with enlarged red circles outlined in black. Major HEV-3 sublineages are highlighted on the phylogeny with clade branch support values shown. The tree was made using RAxML-NG, rooted using a HEV-1a reference sequence (M73218.1) and visualized in R version 4.3.1. The adjacent tile plot depicts RBV resistance-associated mutations: red indicates presence in the majority consensus sequence, and orange indicates minority variants detected at ≥1% prevalence. Clade 1 viruses are shaded in red hues, Clade 2 viruses in blues, subtype 3ra in yellow. Only mutations observed in at least one sample are displayed.

To investigate the phylogeographic context of the Hungarian HEV strains, we performed a phylogenetic analysis using 76 near full-length sequences from both Hungary and other countries (Figure 3). This analysis confirmed the clustering results for all 76 samples, supporting the conclusions drawn from the shorter ORF2-based tree. All near-complete 3a genomes sequenced showed greatest similarity to the French isolate MW355288 with 91–92% identity. Seventeen of them were highly similar and formed a monophyletic group with small pairwise patristic distances and a mean p-distance of 2% on the nucleotide level over the whole genome, excluding the PPR. Hungarian subtype 3c genomes showed more variation with 95–98% similarity to a wide range of isolates from Italy, Germany, France, Spain and the UK. Viruses belonging to subtype 3e formed two distinct groups. Members of the larger group of 3e sequences were 96–99% identical to a virus detected in Italy in the liver of a wild boar (MT840367), or showed 95% identity to a UK isolate (MH504146). Other members were most closely related to French isolate JQ013795, but with only 91–93% similarity. The second group included sequences most similar to Japanese and German strains and also a Hungarian isolate from 2010 (HM055578) with only about 89% identity on the nucleotide level (Figure 3).

Subtype 3f sequences were subclassified into 3f1 and 3f2 by the HEVNET genotyping tool. The division of subtype 3f into these two provisional subgroups was supported by phylogenetic evidence using near-complete genomic sequences (between-group distance of 0.0502). Two 3f1 genomes shared 98–99% identity with isolate MW355330; three others were most closely related to isolates from Spain or Japan. Four subtype 3f2 strains clustered together with Italian wild boar-derived viruses (OK429318, PQ778483 and OK429319), and also with more distantly related sequences with 88–90% identity from Germany (MK089847) and Sweden (KT581444, KT581446, KT581447) (Figure 3).

The two near-complete subtype 3g genomes had a maximum of 85% identity to other genomes in GenBank. The subtype 3h virus belonged to the rare 3h3 subgroup previously only detected in Switzerland, and the subclassification is based on the results of Vonlanthen-Specker et al. [40]. This provisional subgroup is recognized by the HEVNET genotyping tool. The strain showed 96.87% identity with Swiss isolates and was identified in a lung transplant patient with no travel history to Switzerland. The single subtype 3i virus was most similar to a strain found in Sweden (MH377721), but with only 91% identity. The obtained 3m sequence was most closely related to isolate MW355399 from France (Figure 3).

Rabbit-derived 3ra viruses have not been detected in the country before. The two patients with 3ra viruses fell ill in January and May of 2021. The near-complete genome from one patient shared limited resemblance to previously reported sequences, highlighting the extent of uncharacterized variability within this subtype. Only a partial sequence could be obtained from the other patient, but alignment to the near-complete genome showed complete identity.

No subtype could be determined for a patient with a genotype 3 virus using the HEVNET genotyping tool (sample no.: 3062323, Accession no.: PX108708). The strain was most closely related to a Japanese subtype 3a isolate (AB089824) with 88% identity over the whole genome, but it could not be unequivocally assigned to any known subtype and possibly represents a new, unclassified subtype (Figure 3).

HEV genotype 4b was detected in two patients, representing the first documented occurrence of HEV-4 in Hungary. The 348 bp long sequences from ORF2 differed in only one nucleotide position, and the two patients were diagnosed only two weeks apart. However, no geographical or epidemiological connection could be established. One case involved a 38-year-old male from a small village in western Transdanubia, who reported consumption of game meat. The other was a 68-year-old female from Debrecen, the second-largest city in Hungary, located in the eastern part of the country, with no reported exposures. Travel history was unavailable for both patients. Only partial genome sequences could be obtained, but the 1.8 kb sequenced segment from one of the viruses was most closely related to an isolate from Belgium (OM388298).

### 3.2. Detection of Ribavirin Resistance and Analysis of the Proline-Rich Region

The presence of eight mutations associated with ribavirin resistance within the RdRp region of ORF1 was investigated in the sequenced genomes. Mutations Y1320H, K1383N, D1384G, K1398R, and Y1587F were not detected in any of the viruses; however, A723V, G1634R and V1479I were found in the samples (Figure 3). V1479I was detected throughout Clade 1 (30/30, 100%), and was often associated with G1634R in subtype 3e strains (11/13, 84.6%), even though all but one of the samples were collected at the initial detection of the infection. V1479I was found in 4 out of 19 subtype 3c viruses, while G1634R was present in only one. Neither mutation was detected in subtype 3a. Nine further single-nucleotide variations in ORF2 and ORF3 previously shown to appear during ribavirin therapy were also investigated [29]. P95S was found to be widespread and did not seem to be associated with Clades nor with therapy.

**Figure 4:**
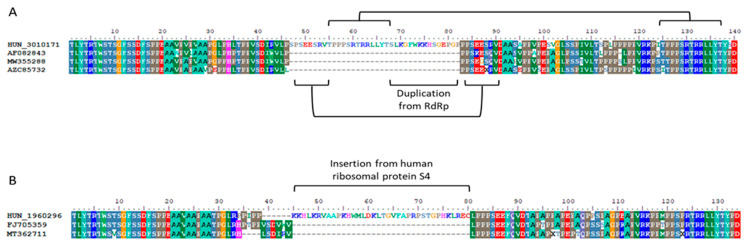
HEV polyproline region amino acid sequences from two patients showing unique insertions and duplica-tions. Sequences were aligned with subtype references (AF082843 for 3a and FJ705359 for 3c) and the most closely related GenBank sequences. (**A**) Subtype 3a from a liver transplant recipient (Patient 13) with a 108 bp insertion comprising duplicated PPR residues and an additional RdRp segment. (**B**) Subtype 3c from a heart transplant recip-ient (Patient 17) with a 36 amino acid insertion from the human ribosomal protein S4.

The proline-rich regions of 76 viruses were analyzed for the presence of insertions, duplications and deletions. The lengths of the PPRs followed the previously described conventions [57]. Two 3f1 viruses exhibited long PPRs with an 87-nucleotide-long insertion, consistent with the findings reported by Lhomme et al. [57]. Unique duplications and insertions were identified in the PPRs of two viruses. Both originated from solid organ transplant recipients with chronic infections. A liver transplant recipient (Table 2, Patient no. 13) infected with subtype 3a received a 3-month course of ribavirin following 3 months of viraemia, resulting in viral clearance from the serum. However, a follow-up sample collected over 3.5 years later revealed a high viral load (1.87 × 10^8^ IU/mL). Sequencing confirmed the presence of a viral strain showing 99% identity with the initially detected strain across 6.1 kbs sequenced at both time points. The PPR included a 108 bp insertion consisting of duplications of 7 and 14 amino acid residues from the PPR, followed by an additional 14 residues from the RdRp region (Figure 4A). The patient received a second course of ribavirin and eventually achieved SVR. The PPR of the subtype 3c sequence obtained from a heart transplant patient (Table 2, Patient no. 17), one year after the initial diagnosis, contained a 36 amino acid segment from the human ribosomal protein S4 with a small deletion of the original sequence at one end of the inserted sequence (Figure 4B).

### 3.3. In Silico Validation of Multiple Infections

**Figure 5:**
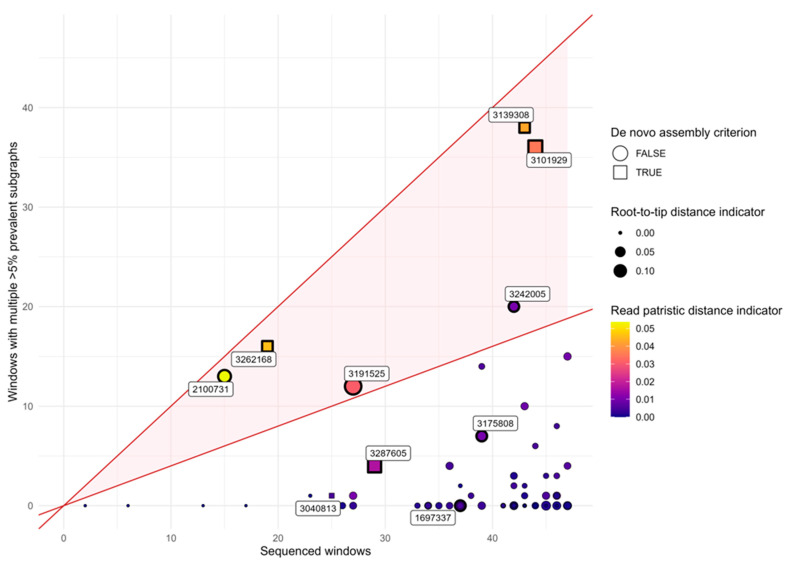
In silico validation of HEV multiple infection. Samples with at least two overlapping but genetically distinct contigs are indicated by rectangles (criterion 1). Points sized and colored according to root-to-tip distance and read patristic distance indicators, respectively, are outlined with a thick stroke if they fall within the top 20% for both indicators (criterion 2). Samples located within the red shaded area have at least 40% of high-quality windows containing multiple sub-graphs, each with at least 5% prevalence (criterion 3). Sample IDs are shown for all cases meeting at least one of these three criteria, and samples meeting at least two are classified as multiply infected.

De novo genome assembly and phyloscanner analyses were used to assess the presence or absence of multiple HEV infections, with three validation criteria applied in combination to minimize the risk of false-positive results arising from sequencing artifacts. Multiple infection with at least two different HEV strains was deemed probable in 6 patients (Figure 5). Two samples (3139308 and 3262168), collected from a heart transplant patient one year apart, both met all three criteria for multiple infection. Subtyping of the de novo assembled contigs indicated that both strains present in the samples belonged to subtype 3c, and both strains persisted. In Sample no. 3101929, which also meets all three criteria, a dominant subtype 3a strain and a minor 3f1 sequence were detected simultaneously. Sample no. 3287605, originating from a cancer patient, met two out of three criteria, and it showed the presence of different subtype 3f2 strains. For three more patients (2100731, 3191525, 3242005), only two criteria were met: mixed infection was suspected based on phyloscanner results, but no sequence belonging to multiple strains could be assembled, probably due to very low copy numbers. Two of these patients received solid organ transplants, and one was pregnant.

### 3.4. Temporal and Geographical Distribution of Detected HEV-3 Subtypes

**Figure 6:**
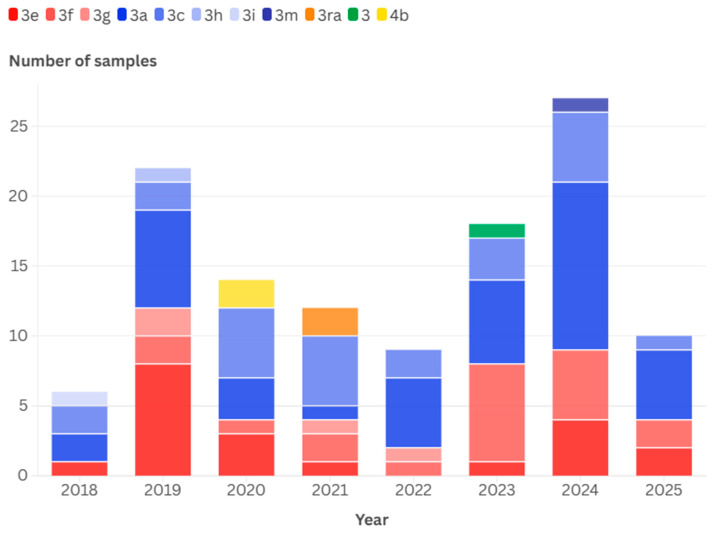
Distribution of HEV-3 subtypes and clades during the study period. Clade 1 viruses are shown in red hues, Clade 2 viruses in blues. Rabbit-derived viruses are shown in orange, 4b subtype viruses in yellow and the single unclassifiable HEV-3 virus in green.

Clade 2 viruses were most common (58.5%) in the study period (Figure 6). Although the number of Clade 1 viruses varied across years, logistic regression analysis did not reveal a significant temporal trend in the proportion of Clade 1 versus Clade 2 across 2018–2025 (β = −0.0535, *p* = 0.541). Of the most common subtypes, the geographical distributions of 3a and 3c were even in all three first-level NUTS regions. Subtype 3e was uncommon in the Great Plain and North region, and subtype 3f was rarely detected in Transdanubia; however, both were prevalent in Central Hungary. As a result of this distribution, Clade 1 viruses were more frequently identified in Central Hungary (25/49, 51.0%) than in Transdanubia (9/40, 22.5%) or the Great Plain and North (9/27, 33.3%). The overall distribution of the clades differed significantly (χ^2^ = 7.29, *p* = 0.026) across regions.

### 3.5. Immunocompromised Patients and Chronic Infections

Out of 48 HEV-positive immunocompromised patients, 20 (20/48, 41.6%) became chronic carriers: 17 SOT recipients, 2 patients with hematological malignancies and 1 cirrhotic patient receiving biological therapy. The distribution of clades did not differ significantly between immunosuppressed and non-immunosuppressed patients. Clade 2 was detected in 64.6% of immunosuppressed patients (31/48) compared with 54.3% of non-immunosuppressed patients (38/70), a difference that was not statistically significant (χ^2^ = 0.86, *p* = 0.355). Infections with Clade 1 and Clade 2 viruses showed similar tendency of progression (χ^2^ = 0.253, *p* = 0.615) to chronic infection (6/16, 38% vs. 14/31, 45%). All chronic HEV patients were treated with ribavirin, and most of them cleared the virus, while four patients are still RNA positive at the time of writing (Table 2). Patient no. 12 underwent three years of ribavirin therapy with several dose adjustments implemented due to anemia, before eventually clearing the virus. The resulting liver damage necessitated a second liver transplantation. Patient no. 13 was treated only three and a half years after initial diagnosis, having been lost to follow-up (also see Section 3.2).

## 4. Discussion

Hepatitis E virus is endemic and notifiable in Hungary, but no seroprevalence data are available for the general population. The incidence of reported cases of HEV infections has tripled in the last decade, increasing from 1.4 in 2014 to 4.2 per 100,000 population in 2024 [58]. The increase may be partly attributed to changing consumption habits, but is more likely explained by the growing awareness of the disease among clinicians. According to the Hungarian Central Statistical Office, the amount of pork consumed annually by the Hungarian population increased from 25.3 to 29.3 kgs per capita in the same period. Game meat is less popular: about 90% of game produced in the country is exported, mainly to Germany and Italy. Data on possible sources of exposure were limited in our study, but based on the above, most HEV infections are assumed to be linked to undercooked pork. However, the role of game meat or blood transfusion cannot be excluded.

Similarly to other reports from Europe, the detected viruses from Hungarian patients almost all belonged to genotype 3. In two previous studies, the analysis of 29 and 20 short sequences of HEV ORF1 and ORF2 obtained from humans, swine, wild boar, and deer before 2010 detected almost exclusively subtype 3a and 3e strains [42,43]. Another Hungarian study, which included 13 strains from human sera collected between 2014 and 2017, showed more variation [44]. In our study, an unusually high level of variation was observed among the detected strains, which included nine different HEV-3 subtypes, an unclassifiable genotype 3 strain, and subtype 4b—found in two patients—marking the first detection of HEV-4 in the country. HEV-4 has been reported only sporadically in Europe, primarily in France, Italy, and Belgium, either in connection with foodborne outbreaks or identified through surveillance studies [6,7,8,9]. While evidence for sustained transmission elsewhere in Europe has not been documented, circulation has been suggested in Italy, where genotype 4d was recently found in wastewater [59]. Although the exact sources are unclear, the detection of multiple HEV-4 subtypes indicates that several independent introduction events have likely occurred over time. The subtype 4b strain detected in our study represents the most prevalent HEV-4 subtype in Europe, and its spread can be anticipated in the future both via trade and cross-border transmission [60]. No HEV-1 cases were detected in our cohort. Travel history was not available for most patients, but the absence of HEV-1 most likely reflects that it has not been established in Hungary, consistent with observations from other European countries.

Increased diversity was expected due to the larger number of genotyped viruses, but other processes and changes were also taking place in the country in the last two decades, possibly affecting subtype diversity. Since joining the European Union, the production of pork in Hungary has declined, but the country has maintained its self-sufficiency. At the same time, both the import and the export of pork increased. The rise in imports has been particularly significant, growing from 66.4 thousand tons in 2004 to 170.4 thousand tons in 2024 [61]. The majority of imported pork came from Germany, Spain, Poland, and the Netherlands, potentially contributing to infections with new HEV subtypes in Hungary [62]. In the early 2000s a shift from Clade 1 to Clade 2 strains was observed in several countries in Western Europe. It was described in Belgium, the Netherlands and later also in Germany [37,63,64]. The previously dominant 3f strains were gradually replaced by Clade 2 viruses, mainly 3c, which is the most prevalent today. A similar trend is only beginning in Spain, where the endemic 3f genotype was almost exclusive in both pigs and humans until 2020, when the increased demand for pork due to the African swine fever pandemic necessitated the import of fattening pigs from other European countries, and so other subtypes appeared in the country [39].

The frequency and subtype distribution of HEV may also have been influenced by the PRRSV (Porcine reproductive and respiratory syndrome virus) eradication program in Hungary, implemented between 2014 and 2022. The program utilized complete herd replacement and the introduction of high-performance breeds to enhance production efficiency and environmental sustainability in the swine sector [65]. Animals were mainly imported from Slovakia, the Netherlands, Germany, and the Czech Republic in this period [62]. In the Czech Republic, data show the dominance of Clade 1 strains, while in Slovakia, one study found subtypes 3i and 3a to be the most common in swine [66,67]. In both the Netherlands and Germany 3c and 3f were found to be dominant in swine populations [37,68]. The gradual repopulation of large-scale fattening and breeding farms with PRRSV-free animals from these countries likely introduced 3c and other HEV subtypes previously not present in swine in the country. Conversely, the process may have also reduced the overall prevalence of HEV infections within the swine population. The potential interaction between HEV and PRRSV has been explored through experimental co-infection studies in specific-pathogen-free pigs [69]. PRRSV is known to impair the immune response, which has been shown to affect HEV infection dynamics, specifically by delaying HEV shedding and increasing the quantity of viral particles shed. Unfortunately, no recent data are available to assess the actual impact of the PRRSV eradication program on HEV prevalence or subtype distribution in Hungarian pig populations. A study among swine could also show whether export from Hungary is affecting the prevalence of different HEV strains in neighboring countries, such as Romania, Croatia, Serbia and Austria. Data from Croatia show the subtype distribution among humans, swine and wild boar to be similar to our study [35]. The authors suggest that 3a and 3c may be endemic in Croatia, while 3e and 3f were more recently introduced by trade or by contact with wild and domestic animals. Evidence for transmission between domestic and wild swine reservoirs also comes from Slovakia, where similar subtype distributions were detected in wild boars and domestic pigs [70]. Moreover, sequence similarities suggested possible cross-border movement of wild boars between Hungary and Slovakia, indicating that animal migration, in addition to swine trade, also shapes subtype distribution in the region.

Interestingly, most of the subtype 3a genomes formed a closely related monophyletic group on the phylogenetic tree. The sequences spanned multiple years and regions, suggesting these genomes may represent a locally established lineage in the country. No such variant cluster was observed for any other subtype. Subtype distributions clearly differed between the three NUTS 1 regions of the country. The Central Hungary region, encompassing the capital and its surrounding metropolitan area, has a total population exceeding 2.9 million, accounting for almost 30% of the Hungarian population. Due to the higher degree of urbanization in this region, it is likely that a greater proportion of the population buys food from supermarket chains and consumes more imported pork and pork products, which might explain the different ratio of the major clades in this area.

Some reports suggest that HEV Clade 2 viruses may be less pathogenic, and the shift in several countries towards these subtypes may lead to fewer patients with severe disease and more asymptomatic cases that are less likely to be diagnosed [64,71]. The changes taking place in Hungary before and during the study period resulted in a balanced distribution of Clade 1 and Clade 2 viruses. More studies are required to confirm the difference in pathogenicity between clades; however, both clades are able to cause chronic infection in immunocompromised patients in similar ratios. HEV testing of immunosuppressed patients in case of elevated liver enzymes is now routinely performed in Hungary, and the high number of these patients in our study highlights the importance of systematic HEV screening in this population. Early diagnosis enables timely initiation of antiviral therapy and closer monitoring of liver function, which may reduce the risk of progressive liver disease. Persistence of HEV was a common occurrence in this patient group, lasting for several years in at least one case, leading to severe liver damage and an escalation of ribavirin-induced side effects. Ribavirin resistance-associated mutations were detected in chronic HEV patients even before treatment and were also present in individuals who successfully cleared the virus, confirming that these mutations can arise naturally and are often associated with subtype.

The risk of zoonotic transmission still needs to be better communicated to vulnerable patients. Prevention strategies should include detailed dietary recommendations to emphasize the avoidance of undercooked pork, wild game, and offal, as these are common sources of zoonotic HEV transmission. High-risk groups would also greatly benefit from the introduction of vaccination, currently not available in Europe.

Lastly, we present a novel bioinformatic approach for detecting dual or multiple HEV infections from next-generation sequencing data. We developed three criteria to indicate unusually high viral diversity of samples: the consistent presence of viral subpopulations along the genome that are not connected by within-host evolution, and the presence of distinguishable lineages through partial genome reconstruction. Despite applying a combined set of complementary and largely independent criteria to detect multiple infections, our approach has limitations. The short reads generated by the Illumina platform constrain the ability of de novo assembly algorithms to reliably distinguish between reads originating from different viral strains, and they limit the robustness of phyloscanner in detecting multiple infections across short genomic windows [23,72]. Moreover, although non-viral reads are filtered out during phyloscanner analysis and laboratory protocols minimize the risk of cross-contamination, such events could theoretically result in false positives. However, the concordance observed between two independent samples, collected one year apart from a chronically infected heart transplant recipient, provides some validation that our bioinformatic approach can distinguish true co-circulating strains from single infection. Globally, data on the prevalence of multiple HEV infections remain limited. However, our findings suggest that such cases are not uncommon, and our proposed bioinformatic framework could contribute to a better understanding of this underrecognized phenomenon.

## 5. Conclusions

The number of reported HEV infections is increasing in Hungary, most likely reflecting enhanced awareness of the disease. Hungary is a small and open economy in Central Europe, deeply integrated into international trade networks, including the import and export of live pigs and pork products. This active trade significantly influences the epidemiology of animal pathogens, including zoonotic hepatitis E virus genotypes. The import of pork and live animals had a profound effect on the observed high diversity of HEV subtypes found in human samples in our study, and resulted in the first identification of genotype 4 and several HEV-3 subtypes previously not detected in the country. Multiple infections and ribavirin-associated mutations were identified in several patients, but their clinical relevance warrants further investigation. Given the key role of immunosuppression in chronic HEV infection and the high proportion of affected immunocompromised patients in our cohort, routine HEV screening and the prevention of zoonotic transmission in this vulnerable population remain essential priorities and ongoing challenges.

## 6. Limitations

Our findings are limited by the lack of patient exposure and travel data, as well as the lack of recent subtype distributions in animal reservoirs, which would be essential to confirm sources and routes of transmission. Samples from Budapest and Pest county were overrepresented in the surveillance. Additionally, limited availability of clinical data on the patients did not allow us to assess differences in pathogenicity across HEV subtypes or clades. Sample storage at −20 °C may have impacted RNA integrity for some samples.

## Figures and Tables

**Table 1:** Primer sets used for Illumina next-generation sequencing.

Primer Name	Sequence	Position on Reference Genome *	Use
15f	5′-TGTGGTCGAYGCCATGGAG-3′	13–31	Amplicon 1
2190r	5′-TCGCTAGARAARCCAGATGTAGACC-3′	2139–2163	
24f	5′-GYTGYTCRCGGCTYATGAC-3′	1032–1050	Amplicon 2
41r	5′-GCCATRTTCCAGACRGTRTTCCA-3′	4628–4650	
123f	5′-AGGGTTGAGCAGAACCC(I)AAGAG-3′	2630–2652	Amplicon 3
20r	5′-GCRAAGGGGTTGGTTGGATG-3′	5340–5359	
126f	5′-TGCCTATGCTGCCCGCGCCACC-3′	5212–5233	Amplicon 4
22r	5′-CCGGGTYTTRCCTACCTTC-3′	7127–7145	
137f	5′-TCTAATGGCYTRGAYTGYACYGC-3′	1892–1914	Sanger sequencing of PPR
124r	5′-ACCGAYGARGCCCGCTGCAT-3′	3182–3201	

* Accession number: FJ705359.

**Table 2:** Details of patients with chronic HEV infections including ribavirin resistance mutations.

Patient No	Sample Number	Age	Gender	Transplanted Organ /Condition	HEV Subtype	Duration of HEV RNA Positivity	Outcome	A723V	G1634R	V1479I	A64T	G38S	P25S	P95S
1	1610753	55.9	f	liver	3a	6 months	cleared	n/a						
2	1623765	62.5	m	heart	3a	8 months	cleared							
3	1635541	64.0	f	liver	3e	3 months	cleared							
4	1665563	64.1	f	kidney	3e	3 months	cleared	n/a						
5	1669232	57.2	m	heart	3a	3 months	cleared							
6	1676451	63.2	m	heart	3f2	3 months	cleared							
7	1686530	69.6	m	liver	3a	6 months	cleared	n/a						
8	1903888	71.2	m	cirrhosis	3c	4 months	cleared							
9	1944665	26.9	f	liver	3a	10 months	cleared							
10	2026372	40.0	f	acute myeloid leukemia	3c	5 months	cleared	n/a						
11	2056231	26.6	m	acute lymphoid leukemia	3e	3 months	cleared							
12	1538390	47.4	f	liver	3c	38 months	cleared							
13	3010171	30.8	f	liver	3a	52 months	cleared							
14	3101306	53.2	f	kidney	3e	5 months	cleared							
15	3141096	45.2	f	kidney	3a	17 months	ongoing							
16	3139308	62.8	f	heart	3c	17 months	ongoing							
17	1960296	37.1	f	heart	3c	12 months	cleared							
18	3238012	42.6	f	heart	3f2	10 months	ongoing							
19	3255840	33.9	m	heart	3m	7 months	cleared							
20	3278671	48.6	m	kidney	3c	5 months	ongoing							

Abbreviations: f = female, m = male, n/a = not available. Gray boxes indicate the presence of RBV-resistance mutation in the majority consensus sequence. White indicates <1% or no mutation detected.

## Data Availability

Sequences obtained during this study were deposited in GenBank under the following accession numbers: PV877305-PV877332, PX108670-PX108747 and PX226753-PX226754. The raw sequencing data generated in this study are available in the NCBI Sequence Read Archive (SRA) under BioProject accession number PRJNA1327628.

## Abbreviations

The following abbreviations are used in this manuscript:

HEV	Hepatitis E virus
HVR	Hypervariable Region
NUTS	Nomenclature of Territorial Units for Statistics
ORF	Open reading frame
PPR	Polyproline region
SOT	Solid organ transplant
SVR	Sustained viral response
RdRp	RNA-dependent RNA polymerase
RIVM	Rijksinstituut voor Volksgezondheid en Milieu
RNA	Ribonucleic Acid
RT-PCR	Reverse Transcription Polymerase Chain Reaction
WHO	World Health Organization
